# Association of maternal smoking during pregnancy with youth depression and subsequent adult chronic diseases in offspring

**DOI:** 10.1038/s41398-026-03976-w

**Published:** 2026-03-26

**Authors:** Wenming Wei, Bolun Cheng, Xin Qi, Dan He, Shiqiang Cheng, Xuena Yang, Chuyu Pan, Peilin Meng, Jingni Hui, Yifan Gou, Boyue Zhao, Li Liu, Yan Wen, Huan Liu, Yumeng Jia, Feng Zhang

**Affiliations:** 1 Key Laboratory of Trace Elements and Endemic Diseases of National Health and Family Planning Commission, Key Laboratory of Environment and Genes Related to Diseases of Ministry of Education of China, Key Laboratory for Disease Prevention and Control and Health Promotion of Shaanxi Province, School of Public Health, Health Science Center, Xi’an Jiaotong University, Xi’an, China; 2 Department of Psychiatry, the First Affiliated Hospital of Xi’an Jiaotong University, Xi’an, P. R. China

**Keywords:** Genomics, Depression

## Abstract

This study aimed to elucidate the relationship between maternal smoking during pregnancy (MSDP) and the incidence of youth depression, examine the modifying role of genetic susceptibility, and assess subsequent physical health risks in adulthood. We utilized data from 60,839 participants in the UK Biobank. Cox proportional hazards models were applied to evaluate the impact of MSDP on the onset of youth depression. Genome-wide association studies (GWAS) and gene–environment interaction analyses were conducted to identify genetic variants influencing youth depression and their interactions with MSDP. Polygenic risk scores (PRS) were calculated to assess genetic contributions. Multi-state modeling explored health transitions from MSDP exposure to youth depression and 24 chronic physical diseases in adulthood. The GWAS identified 10 significant SNPs within the *ABR* gene region (*P* < 5 × 10^−8^) associated with youth depression. MSDP was associated with higher risks of youth depression (HR = 1.26, 95% CI: 1.18–1.35), especially among females (HR = 1.34, 95% CI: 1.24–1.45) and participants with high PRS (HR = 2.35, 95% CI: 1.97–2.80). Additionally, MSDP was associated with increased risks of five chronic conditions, including asthma (HR = 1.37, 95% CI: 1.14–1.66), chronic obstructive pulmonary disease (COPD) (HR = 3.53, 95% CI: 2.20–5.65), hypertension(HR = 1.29, 95% CI: 1.12–1.50), liver disease (HR = 1.88, 95% CI: 1.31–2.71), and peripheral vascular disease (HR = 2.33, 95% CI: 1.47–3.71). Notable gender differences in these effects were observed. Overall, MSDP was associated with a greater likelihood of youth depression, especially among females and those with higher genetic susceptibility, and with a higher burden of chronic physical conditions in adulthood. Targeted smoking cessation during pregnancy may therefore yield substantial intergenerational benefits for both mental and physical health.

## Introduction

Globally, one in seven adolescents aged 10 to 19 experiences a mental disorder, which represents 13% of the global burden of disease in this age group (World Health Organization, 2021). Depression ranks as the second most prevalent mental disorder among children and adolescents [1]. Over the past decade, the incidence of depression among adolescents has risen significantly, especially in females [2]. Gender differences in major depression emerge around age 12 and continue into adulthood, with a peak around ages 13 to 15 [3]. Youth depression not only seriously affects social functioning [4] but also frequently co-occurs with other mental illnesses, potentially leading to severe physical and mental health issues in adulthood [5, 6], such as cardiovascular diseases [7] and obesity [8]. Furthermore, individuals with severe mental disorders exhibit a higher prevalence of multiple physical comorbidities [9]. Therefore, the early identification and management of risk factors for youth depression are critical for effective prevention.

Maternal smoking during pregnancy (MSDP) is an environmentally controllable factor. Despite public health efforts that have reduced its prevalence [10], MSDP still affected 250 million women worldwide in 2020, maintaining a global rate of 1.7% [11]. The relationship between MSDP and youth depression, however, remains elusive. Research suggests that MSDP is linked to severe emotional and behavioral disorders in adolescents [12], as well as anxiety and depressive behaviors in children aged 18 to 36 months [13]. Contrarily, another study found no significant association between heavy MSDP and depression in offspring [14]. These contradictory results underscore the necessity of conducting a large-scale prospective cohort study to delve deeper into this relationship. Moreover, existing studies have primarily concentrated on the direct effects of MSDP or genetic variants on youth depression, largely overlooking the potential interactions between genetic and environmental factors and their combined effects.

Understanding the interplay between MSDP and genetic susceptibility is crucial, but progress has been hindered by limited knowledge of the genetic architecture of youth depression itself. Compared to adult depression, youth depression exhibits significant heterogeneity, marked by noticeable differences in brain structure [15], heritability [16], and treatment responses [17]. Twin studies have also shown genetic variations between childhood and adult depression [18]. While large-scale genome-wide association studies (GWAS) have provided substantial insights into the genetic underpinnings of adult depression [19], youth-focused GWAS research is conspicuously absent. We conducted a GWAS on youth depression, complemented by a gene-environment interaction study on MSDP. Additionally, polygenic risk scores (PRS) have proven effective in quantifying the cumulative effects of multiple risk-related variants [20], facilitating the exploration of the combined impacts of environmental exposures and genetic factors on depression [21].

In this study, we conducted a prospective study utilizing the UK Biobank to explore the association between MSDP and youth depression, as well as the interplay and combined impact of MSDP exposure and genetic factors on the onset of youth depression. Finally, we traced the continuum of health challenges among youth exposed to MSDP—from early-life exposure and the onset of depression to the development of chronic physical diseases and mortality—aiming to provide a comprehensive understanding of the life-course health consequences of this exposure.

## Methods

### Study population

This study employed data from the UK Biobank [22], which contains health and genetic profiles from approximately 500,000 individuals, aged 40 to 69 at recruitment, collected between 2006 and 2010. About 50,000 samples were first genotyped using the UKBiLEVE Axiom array, and the remaining were analyzed with the UK Biobank Axiom array by Affymetrix, targeting approximately 800,000 single nucleotide polymorphisms (SNPs). Principal component analysis was applied to manage population stratification for about 500,000 samples, focusing on 100,000 SNPs. Post extensive quality control, the refined dataset included 670,739 autosomal markers for 487,442 individuals, with data prephasing and imputation conducted using SHAPEIT3 and IMPUTE4, resulting in a comprehensive dataset of nearly 96 million variants for 487,411 participants.

**Fig. 1:**
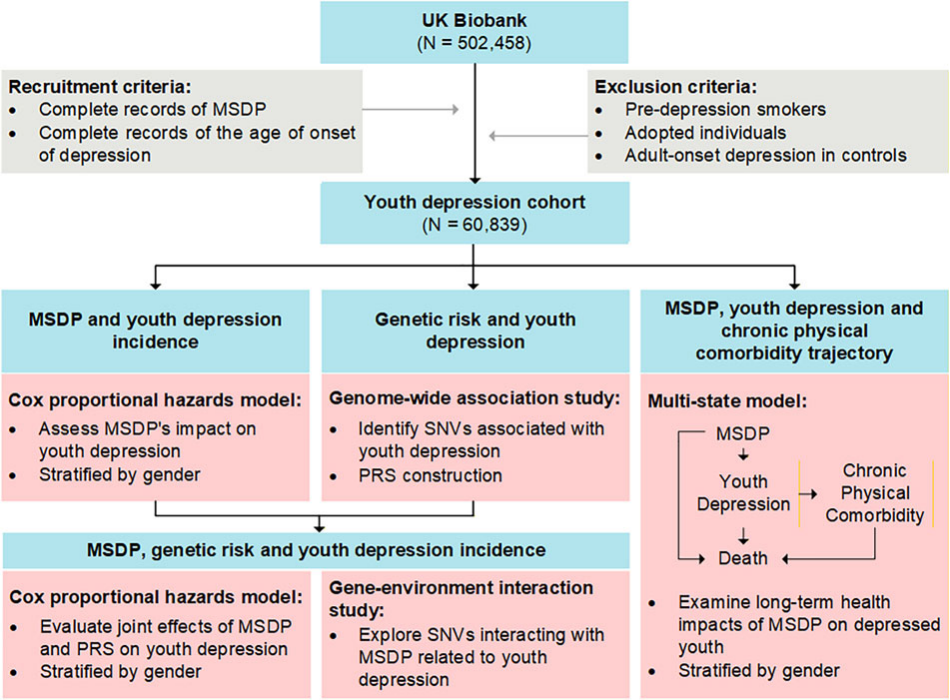
Flowchart of Study Design and Methodology. MSDP maternal smoking during pregnancy.

The analysis targeted a subset of data related to MSDP, depression, and chronic diseases. The inclusion criteria required complete records of maternal smoking at birth and documented ages of onset for depression. Individuals lacking essential data, lost to follow-up, who began smoking before youth depression onset, or were adopted were excluded. The design and methodology of this study are illustrated in Fig. 1.

### Primary variable definitions

This study defined youth depression using responses from Data-Field 20433, which inquired about the age at the first depressive episode lasting two weeks or longer. Cases of youth depression included participants who reported their first such episode before turning 18 and answered “Yes” to either Data-Field 20446, regarding periods of feeling sad, blue, or depressed for two weeks or more, or Data-Field 20441, about losing interest in typically enjoyable activities for a similar duration. The control group comprised participants who did not meet these criteria. To reduce bias, individuals in the control group who reported adult-onset depression were excluded. These cases were identified using code 1286 from Data-Field 20002, codes 3, 4, or 5 from Data-Field 20126, and code 11 from Data-Field 20544, ensuring an accurate comparison between youth depression cases and controls.

MSDP was evaluated via Data-Field 1787, which asked, “Did your mother smoke regularly around the time when you were born?” Responses such as “Do not know” and “Prefer not to answer” were excluded. To control for confounding factors associated with smoking behaviors, individuals who began smoking before the onset of youth depression were excluded, based on their recorded age at smoking initiation in Data-Field 3436.

We analyzed 24 physical health outcomes based on the Charlson and Elixhauser comorbidity indices [23, 24]. These conditions encompass a wide range, including asthma, chronic obstructive pulmonary disease (COPD), cardiac arrhythmia, congestive heart failure, myocardial infarction, cerebrovascular disease, various neurological disorders (such as epilepsy, multiple sclerosis, Parkinson’s disease, and seizures, excluding cerebrovascular disease and dementia), cancer, both types of diabetes, hypothyroidism, liver disease, renal disease, peptic ulcers, rheumatic and collagen diseases, paralysis or paresis, HIV/AIDS, hypertension, peripheral vascular disease, pulmonary circulation disorders, valvular disease, deficiency anemia, blood loss anemia, coagulopathy, and fluid or electrolyte disorders. To comprehensively evaluate the progression of these conditions, five new indicators were developed to measure the time to onset of an individual’s nth (*n* = 1 to 5) comorbidity. This approach allows for a detailed analysis of the cumulative burden of maternal smoking and youth depression on chronic disease progression. The ICD-10 codes for these 24 conditions are detailed in Supplementary Table 1.

### Covariate definitions

Gender was defined according to participants’ demographic records. Ethnic background was categorized as “White” or “Non-White” based on Data-Field 21000. Birth weight (in grams) was obtained from Data-Field 20022. Breastfeeding status was derived from Data-Field 1677, and comparative body size at age 10 was assessed using Data-Field 1687. Smoking status (ever vs. never) was obtained from Data-Field 20160, and alcohol consumption (ever vs. never) was defined using Data-Field 20117.

### Statistical analysis methods

#### Cox regression analysis

We conducted a Cox proportional hazards regression analysis to evaluate the impact of MSDP on the onset of youth depressive symptoms. To assess sex-specific effects, we included a multiplicative MSDP × sex interaction term in the model and further evaluated results in sex-stratified analyses. Covariates included gender, birth weight, ethnic background, breastfeeding status, and comparative body size at age 10. The proportional hazards assumption was verified using Schoenfeld residuals, confirming the model’s appropriateness. Statistical significance was set at a *p*-value threshold of less than 0.05. All analyses were performed using the “*survival*” package in R 4.3.0.

Cumulative incidence curves were generated to illustrate the time-dependent risk of youth depression by MSDP exposure. Age at onset was used as the time-to-event variable, and participants without depression were censored at the end of follow-up. Nonparametric survival estimates were obtained using the Kaplan–Meier method and visualized in *ggplot2* (version 3.5.2).

#### Genome-wide association study and gene-environment interaction analysis

We performed a genome-wide association study (GWAS) using PLINK 2.0 [25] to investigate the genetic basis of youth depression. Covariates included gender, birth weight, ethnic background, breastfeeding status, body size at age 10, and the first 10 principal components to adjust for population stratification. Quality control criteria were stringent, setting a minor allele frequency (MAF) threshold at 0.01, genotype missingness for individuals and SNPs at no more than 10%, and a Hardy-Weinberg equilibrium (HWE) *p*-value at 0.001. The genome-wide significance threshold was established at a *p*-value of less than 5 × 10^−8^.

Linkage Disequilibrium Score Regression (LDSC) was performed to estimate the total observed scale heritability (*h*^2^) for youth depression. Additionally, we conducted a genetic correlation analysis between youth depression and adult depression. For this analysis, we obtained GWAS summary statistics for adult depression from the Psychiatric Genomics Consortium (PGC). LDSC was then utilized to estimate the genetic correlation (*r*_*g*_) between the two phenotypes.

Subsequently, we conducted a gene-environment interaction analysis using PLINK 2.0 to explore the interplay between genetic variants and MSDP on youth depression. This analysis adhered to the same covariates and quality control standards as the GWAS. We set the genome-wide significance threshold at a *p*-value below 5 × 10^−8^ and employed a more relaxed threshold of 1 × 10^−5^ to identify potential gene-environment interactions.

#### Polygenic risk score analysis

PRS were calculated using our GWAS results via the PRSice-2 software [26], focusing on individuals of White ethnicity and adjusting for gender and the first 10 principal components. PRSice-2 automatically identified the most informative threshold, selecting a *p*-value of 1 as optimal and yielding a robust *R*^2^ of 0.566.

We then investigated the association between MSDP and genetic susceptibility to youth depression by assessing the impact of MSDP on standardized PRS (z-score normalization). This analysis utilized a linear model, adjusting for sex, birth weight, body size at age 10, and breastfeeding history. Subsequently, participants were stratified into low, intermediate, and high genetic risk groups based on tertiles of the control group’s PRS. Within each risk group, Cox proportional hazards regression analyses were performed to assess the effects of MSDP on the onset of youth depression. These analyses retained adjustments for the aforementioned covariates and included stratification by gender to investigate potential sex-specific effects.

#### Estimation of population attributable risks

We estimated the population attributable risks (PARs) and 95% confidence intervals (CIs) to assess the proportion of youth depression cases that could potentially be prevented by eliminating MSDP. These estimates were computed for both the total population and stratified subpopulations based on varying levels of genetic risk and gender. The PAR was calculated using the following established epidemiological formula: *PAR*_*i*_ = *P*_*i*_ × (*HR*_*i*_ − 1)/(*P*_*i*_ × (*HR*_*i*_ − 1) + 1), where *P*_*i*_ represents the prevalence of exposure and *HR*_*i*_ denotes the hazard ratio for the exposed group (*i*) relative to the unexposed, which was used as an approximation of the relative risk (RR) [27]. This formula reflects the proportion of cases in the population that can be attributed to the exposure, assuming a causal relationship. The 95% CIs for the PARs were derived using a simulation-based approach.

#### Multi-state analysis

We employed a multi-state model to assess the impact of MSDP on health transitions among youth depression, chronic physical comorbidities, and mortality. The model identified five critical transitions: from MSDP to youth depression, from MSDP to death, from youth depression to chronic physical illness, from youth depression to death, and from chronic physical illness to death, all of which are depicted in Fig. 1. The model accounted for covariates including sex, birth weight, ethnic background, breastfeeding status, and comparative body size at age 10. For transitions that represent long-term adult physical health processes, we additionally adjusted for adulthood smoking status and alcohol consumption. Results are presented as HRs and 95% CIs. Statistical significance for the transitions involving 24 chronic physical comorbidities was determined using a Bonferroni correction, setting the *p*-value threshold at 0.0021 (0.05/24). A standard significance threshold of 0.05 was applied for the five newly developed indicators. All analyses were conducted using the “*mstate*” package in R 4.3.0.

## Results

### Baseline characteristics

The characteristics of the study participants are detailed in Table 1. The proportion of MSDP exposure was higher in youth with depression (31.98%) compared to their non-depressed counterparts. Additionally, the representation of males was lower among youth diagnosed with depression (32.12%).

**Table 1:** Characteristics of the study population.

	Youth depression cohort (*n* = 60839)	
	Case	Control
Number of participants	6588	54,251
Maternal smoking around birth (%)	2107 (31.98)	14,642 (26.99)
Male (%)	2116 (32.12)	29,176 (53.78)
White race (%)	6336 (96.17)	52,443 (96.67)
Birth weight, kg	3.33	3.37
Missing value	2196	21,369
Body size at age 10 year (%)		
Thinner	2272 (34.49)	17,190 (31.69)
Plumper	1233 (18.72)	7672 (14.14)
About average	3041 (46.16)	28,764 (53.02)
Missing value	42	625
Breastfed as a baby (%)	3894 (59.11)	33,248 (61.29)
Missing value	1037	10,509

### Impact of maternal smoking during pregnancy on youth depression risk

**Fig. 2:**
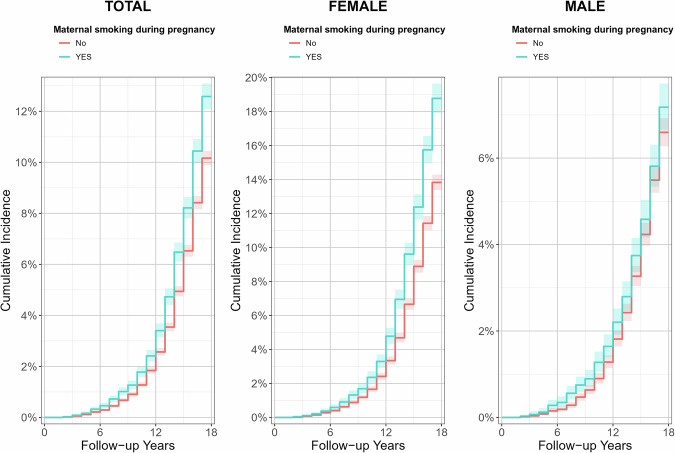
Cumulative incidence curves for youth depression by maternal smoking during pregnancy exposure and gender. From left to right, the panels show the total sample, females, and males. Red curves indicate participants not exposed to maternal smoking during pregnancy, and turquoise curves indicate exposed participants. The *x*-axis shows follow-up time (years), and the *y*-axis shows cumulative incidence of youth depression.

Individuals exposed to MSDP exhibited a higher cumulative incidence of youth depression compared to their non-exposed counterparts. This disparity in cumulative incidence was more pronounced among females than males. Notably, the incidence of youth depression began to increase significantly at around 6 years of age and continued to rise steeply through 18 years, as illustrated in Fig. 2.

MSDP was associated with an increased risk of youth depression (HR = 1.26, 95% CI: 1.18–1.35). A significant MSDP × sex interaction was observed (HR = 0.79; 95% CI: 0.68–0.93), indicating differential effects by sex. In sex-stratified analyses, the association was stronger in females (HR = 1.34, 95% CI: 1.24–1.45) and not statistically significant in males. Our model met the proportional hazards assumption. Detailed findings are presented in Table 2, and the PH assumption test results are depicted in Supplementary Figure 1.

**Table 2:** Association between maternal smoking during pregnancy and risk of youth depression by gender.

Subgroup	Model 1			Model 2		
	Case/Control	HR (95% Cl)	*P*	Case/Control	HR (95% Cl)	*P*
Total	6588/54,251	1.26 (1.20–1.32)	< 2 × 10^−16^	3996/29,719	1.26 (1.18–1.35)	2.53 × 10^−11^
Female	4472/25,075	1.40 (1.32–1.49)	< 2 × 10^−16^	2917/16,007	1.34 (1.24–1.45)	4.06 × 10^−13^
Male	2116/29,176	1.09 (1.00–1.20)	0.0619	1079/13,712	1.06 (0.93–1.21)	0.372

Model 1: Adjusted for gender only. Model 2: Adjusted for multiple covariates including gender, birth weight, ethnic background, breastfeeding status, and comparative body size at age 10.

### GWAS findings and gene-environment interactions in youth depression

**Fig. 3:**
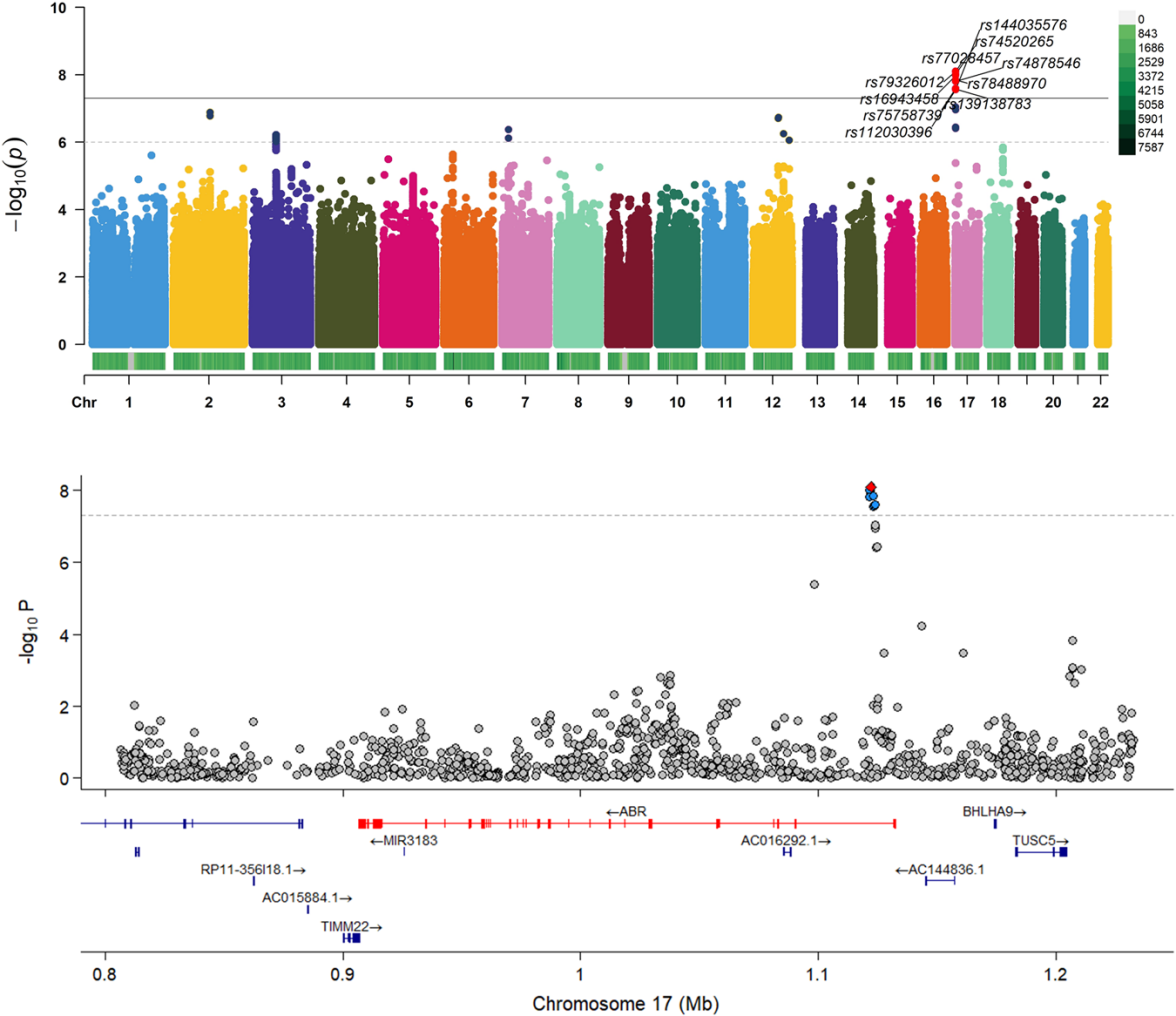
GWAS results identifying significant SNPs in youth depression. The upper plot shows a Manhattan plot highlighting significant SNPs across all chromosomes. The *y*-axis represents the negative logarithm of the *P* values and the *x*-axis represents each chromosome. SNPs surpassing the genome-wide significance threshold (*P* < 5 × 10−8) are highlighted and labeled with their respective rs numbers. The lower plot shows a detailed regional plot of the ABR gene on chromosome 17, illustrating the specific location of SNPs relevant to their association with youth depression.

GWAS identified ten novel significant SNPs associated with youth depression, all located within the *ABR* gene on chromosome 17 (Fig. 3A). A regional association plot provides a detailed view of this critical area, pinpointing the precise locations of these SNPs (Fig. 3B). Detailed information on these ten SNPs is available in Supplementary Table 2. For female, our analysis revealed a total of fourteen significant SNPs, including the previously mentioned ten, all located within the same *ABR* gene on chromosome 17. No significant loci were detected in male adolescents. Gender-specific GWAS results are presented in Supplementary Figure 2.

The heritability (*h*^2^) for youth depression was 0.105 (standard error = 0.020). The genetic correlation (*r*_*g*_) between youth depression and adult depression was 0.644 (standard error = 0.079, *P* = 3.63 × 10^−16^). This result revealed a significant but incomplete genetic overlap between the two phenotypes, suggesting that the genetic foundations of youth and adult depression may have unique components.

Our analysis identified 14 SNPs that potentially interact with MSDP, each with a *p*-value below 1 × 10^−5^. These SNPs are primarily located on chromosomes 6, 8, and 18. Notable genes impacted include *PDE10A*, *DYNLT2B*, *LZTS1*, *LINC02456*. Detailed information is provided in Table 3.

**Table 3:** Detailed information on SNPs interacting with maternal smoking during pregnancy.

SNP	Chr:pos	A1	ALT	OR	95% CI	*P*	Annotation	Nearest gene
rs9459419	6:165867911	C	T	0.88	0.83–0.93	1.79 × 10^−06^	intronic	*PDE10A*
6:165870833_GT_G	6:165870833	G	GT	0.88	0.83–0.93	1.82 × 10^−06^	-	*-*
8:110816254_ATC_A	8:110816254	A	ATC	1.15	1.08–1.22	2.15 × 10^−06^	-	*-*
rs1849204	6:165873851	A	G	0.88	0.83–0.93	2.21 × 10^−06^	intronic	*PDE10A*
rs4490643	6:165880145	A	G	0.81	0.74–0.89	2.37 × 10^−06^	intronic	*PDE10A*
rs4259393	8:110825738	G	C	1.14	1.08–1.20	2.63 × 10^−06^	intronic	*-*
rs9459420	6:165870198	A	G	0.88	0.83–0.93	2.77 × 10^−06^	intronic	*PDE10A*
rs7748041	6:165871066	T	C	0.88	0.83–0.93	3.05 × 10^−06^	intronic	*PDE10A*
rs1542624	8:110817650	C	T	1.14	1.08–1.20	3.37 × 10^−06^	-	*-*
rs7844635	8:110820713	C	A	1.14	1.08–1.20	3.38 × 10^−06^	-	*-*
rs1484017	8:110822888	C	T	1.14	1.08–1.20	5.00 × 10^−06^	-	*-*
rs112796437	3:196045846	G	A	1.14	1.08–1.21	5.18 × 10^−06^	intronic	*DYNLT2B*
rs6914589	6:165868289	G	A	0.89	0.84–0.94	5.62 × 10^−06^	intronic	*PDE10A*
rs13259502	8:20125007	T	A	1.17	1.09–1.25	5.99 × 10^−06^	intronic	*LZTS1*
rs221753	6:165856021	A	G	0.84	0.78–0.91	6.28 × 10^−06^	intronic	*PDE10A*
6:165879446_AAG_A	6:165879446	A	AAG	0.81	0.74–0.89	6.79 × 10^−06^	-	*-*
rs4528676	18:57457497	C	G	1.20	1.11–1.30	7.97 × 10^−06^	-	*-*
rs10745943	12:102980756	C	A	1.20	1.11–1.30	8.83 × 10^−06^	intronic	*LINC02456*

### Joint analyses of maternal smoking during pregnancy and PRSs with youth depression

Participants with higher PRS exhibited a significantly increased prevalence of youth depression (OR = 7.25, 95% CI: 6.87–7.69). The PRS for youth depression are associated with exposure to MSDP (OR = 1.11, 95% CI: 1.07–1.15).

We observed joint effects of MSDP exposure and high PRSs on youth depression. Specifically, participants with high PRSs exposed to MSDP demonstrated a hazard ratio (HR) of 2.35 for the incidence of youth depression (95% CI: 1.97–2.80). Notably, the association was stronger among females with high genetic risk (HR = 1.22, 95% CI: 1.12–1.33), whereas no significant association was observed among their male counterparts. Detailed information is available in Table 4.

**Table 4:** Joint effects of maternal smoking during pregnancy and PRS on youth depression incidence by gender.

Subgroup	Genetic Risk	case/*n*	Standard Error	HR (95% Cl)	*P*
Total	High	3179/8735	0.039	1.16 (1.08–1.25)	8.78 × 10^−05^
	Intermediate	92/8767	0.225	1.36 (0.87–2.12)	0.182
	Low	33/11,772	0.393	1.12 (0.52–2.41)	0.778
Female	High	2281/4078	0.045	1.22 (1.12–1.33)	1.18 × 10^−05^
	Intermediate	62/4024	0.271	1.48 (0.86–2.55)	0.156
	Low	24/7839	0.482	1.19 (0.47–2.99)	0.71
Male	High	898/4657	0.075	1.03 (0.89–1.19)	0.703
	Intermediate	30/4743	0.402	1.11 (0.49–2.50)	0.799
	Low	11/3901	0.682	1.07 (0.30–3.88)	0.913

### PAR estimates for youth depression incidence

We calculated the PAR for youth depression attributable to MSDP. The overall population PAR was 6.03% (95% CI: 4.69%-7.28%). For individuals with high PRS, the PAR was 4.03% (95% CI: 2.36%-5.59%). For female youth, the PAR was higher at 7.26% (95% CI: 5.81%-8.61%), and for those with high PRS, it was 5.16% (95% CI: 3.36%-6.82%). These findings suggest that reducing MSDP could substantially decrease the incidence of youth depression, particularly among females and even in individuals with a high genetic risk.

### Chronic health implications of maternal smoking during pregnancy for depressed youth

Exposure to MSDP was associated with increased risks of youth depression (HR = 1.26, 95% CI: 1.18–1.35) and mortality (HR = 1.55, 95% CI: 1.23–1.96). Additionally, it was associated with higher risks of five specific chronic physical conditions with depressed youth: asthma (HR = 1.37, 95% CI: 1.14–1.66), COPD (HR = 3.53, 95% CI: 2.20–5.65), hypertension (HR = 1.29, 95% CI: 1.12–1.50), liver disease (HR = 1.88, 95% CI: 1.31–2.71), and peripheral vascular disease (HR = 2.33, 95% CI: 1.47–3.71). Moreover, exposure to MSDP also increased the cumulative risk of developing one to four comorbid chronic conditions among these youth. Detailed information is provided in Supplementary Tables 3.

For female offspring, MSDP exposure was not only associated with an increased risk of youth depression (HR = 1.34, 95% CI: 1.24–1.45) but also with an increased prevalence of two specific chronic diseases in youth with depression: COPD (HR = 3.16, 95% CI: 1.85–5.40) and hypertension (HR = 1.32, 95% CI: 1.11–1.58). Furthermore, in female youth with depression, MSDP exposure raised the cumulative risk of developing one to five comorbid chronic conditions. In male youth with depression, MSDP exposure was associated with an increased risk of developing COPD (HR = 5.10, 95% CI: 1.88–13.84) and with a higher cumulative burden of one or three comorbid chronic conditions. Detailed gender-specific results are presented in Supplementary Tables 4 and 5.

## Discussion

To our knowledge, this is the first study that identified an increased risk of developing youth depression associated with exposure to MSDP. Genetic predispositions also play a significant role, as individuals with high genetic risks are more susceptible to youth depression under MSDP exposure. We identified ten SNPs associated with youth depression and an additional fourteen SNPs that may interact with MSDP exposure, potentially influencing susceptibility to this condition. Moreover, MSDP exposure is associated with an elevated risk of various chronic physical health problems in youth with depression. Notable gender differences in these effects were also observed.

Mechanisms underlying youth depression differ significantly from those in adult depression, necessitating targeted research. We conducted the first GWAS of youth depression, characterized by its largest sample size encompassing the entire age spectrum of this group. We identified ten novel SNPs mapping to *ABR* (activator of RhoGEF and GTPase), a bifunctional Rac/Rho GTPase regulator enriched at excitatory synapses through its interaction with PSD-95, positioning it within postsynaptic compartments that orchestrate actin-dependent plasticity [28]. Although no human evidence currently implicates *ABR* directly in mood phenotypes, its functional profile converges with synaptic and stress-responsive mechanisms repeatedly linked to depression. *ABR* knockout increases basal Rac1 activity yet disrupts hippocampal long-term potentiation (LTP) and recognition memory, indicating that *ABR* is required to appropriately constrain Rac1 signaling for durable synaptic strengthening [28]. This function aligns with pathways central to internalizing risk—including Rac1/Cdc42-driven spinogenesis and AMPAR clustering [29], and the stress sensitivity of Rac1 signaling shaping neurodevelopmental and plasticity responses [30]. Additional translational plausibility comes from studies of *BCR*, the *ABR* paralog, where perturbation alters Rac1 activity and aversive learning [31]. These convergent synaptic and stress-responsive mechanisms nominate *ABR* as a biologically coherent contributor to youth depression vulnerability, and the locus represents a direction for LD-aware fine-mapping and targeted circuit-level perturbation studies to clarify how *ABR* may shape mood-relevant development.

In our study, we found that MSDP exposure could significantly increase the risk of youth depression incidence, with an estimated 6.03% of depression cases potentially preventable if MSDP is avoided. This finding aligns with the findings of Ashford et al., who found that exposure to MSDP in a sample of 397 individuals was linked to an increased likelihood of developing internalizing symptoms from ages 5 to 18 years [32]. Nicotine, the primary psychoactive component of tobacco, is known to cross the placental barrier, interacting with receptors that are abundant and actively developing in the fetal brain [33, 34], thereby potentially influencing brain development and subsequent behavioral outcomes. Not only does MSDP lead to observable reductions in cerebral gray and white matter and gyrification in ten-year-old children [35], but it also correlates with behavioral and emotional problems in younger children, evidenced by reduced volumes in key brain regions like the caudate and nucleus accumbens and cortical thinning in the frontal and parietal areas [36]. Furthermore, prenatal nicotine critically impairs neurodevelopment, particularly affecting the hindbrain and midbrain. It disrupts functions in the pedunculopontine tegmental nucleus (PPTg), essential for arousal and motivational responses [37], and alters dopamine systems by modifying release mechanisms in the ventral tegmental area (VTA) [38]. Electrophysiological evidence also shows that such exposure reduces intracellular calcium and glutamate responses in laterodorsal tegmental (LDTg) neurons [39], impacting NMDA receptor-mediated signaling [40]. These neurochemical alterations are likely contributors to the behavioral changes observed in youth.

In addition to the direct harmful effects of cigarette components, the interaction between MSDP and genetic factors may jointly contribute to an increased susceptibility to youth depression. Our findings indicate that MSDP exposure significantly increases the risk among genetically susceptible individuals, potentially preventing up to 4.03% of youth depression cases by avoiding MSDP. Although studies on the combined effects of MSDP and genetic susceptibility in youth depression are limited, available evidence points to a significant genetic component influenced by MSDP, typically explained through epigenetic mechanisms [41]. Our research explored genetic variations and identified several genes that may interact with MSDP in youth susceptible to depression, including *PDE10A, LZTS1*, *DYNLT2B*, and *LINC02456*. These genes are involved in intracellular signaling, gene expression regulation, and neurodevelopmental disorders [42–44]. Notably, we found that genetic susceptibility to youth depression is linked to MSDP exposure. This gene-environment interaction suggests that individuals with higher genetic loading are more likely to encounter MSDP, resulting in a compounded risk effect.

Our study revealed that MSDP greatly escalated the risk of five chronic physical comorbidities in depressed youth, namely asthma, COPD, hypertension, peripheral vascular disease, and liver disease. And, MSDP exposure increased the cumulative risk of developing multiple comorbid chronic conditions among depressed youth. Previous studies have confirmed that MSDP exposure increases the risk of developing youth depression and these chronic physical conditions [45–49], but how MSDP affects disease transition is unclear. Given that depression itself is linked with an increased risk of chronic physical illnesses [50], we hypothesized that MSDP may intensify comorbid mechanisms through two main pathways: behavioral and inflammatory. Individuals with youth depression frequently engage in behaviors such as smoking and drinking [2], thereby exacerbating risks for asthma [51], COPD [52], hypertension [53], and peripheral vascular diseases [54]. Similarly, alcohol consumption further increases the risk of circulatory system disorders and liver diseases [55]. Depression is also associated with elevated inflammatory levels [56], which may trigger these same conditions [51, 57–60]. Well-documented evidence supports the connection between MSDP and these pathways, showing that exposure leads to increased smoking habits [61], nicotine dependence [62], and a higher lifelong risk of alcohol abuse among offspring [63]. Furthermore, MSDP causes enduring inflammatory alterations in newborns, resulting in enduring effects [64]. Although these findings underscore significant correlations, further research is necessary to confirm these results and elucidate the underlying mechanisms.

We observed that females exposed to MSDP showed a higher vulnerability to youth depression than males, consistent with the well-established sex difference in depressive disorders [2] and with prior sex-stratified GWAS demonstrating a stronger polygenic burden in females [65]. Several biologically grounded mechanisms may contribute to this sex-specific susceptibility. First, prenatal hormonal environments differ substantially by fetal sex: males are exposed to higher intrauterine testosterone, which may confer partial neurodevelopmental protection, whereas females lack this protective hormonal milieu, potentially increasing sensitivity to neurotoxic exposures such as cigarette smoke [66, 67]. Evidence from twin studies further supports the influence of prenatal testosterone exposure on later internalizing symptoms [66]. Second, sex differences in placental gene regulation mean that male and female fetuses receive distinct maternal inflammatory and endocrine signals [68]. Third, prenatal smoke exposure can alter testosterone profiles in females [69], and absolute serum testosterone levels have been associated with depressive symptoms in women [70]. In addition, sex-dependent neurodevelopmental responses to smoking have been reported, including differential effects on brain regions involved in emotion regulation [71]. These combined biological, physiological, and hormonal factors likely contribute to the heightened vulnerability to depression observed in young females following MSDP exposure.

Our study represents a discovery-stage GWAS and the largest investigation to date of youth depression, providing a comprehensive genetic perspective. It is also among the first to assess the effects of MSDP on the incidence of youth depression and chronic physical comorbidities in adulthood, providing vital public health insights into the toxic effects of early tobacco exposure. We also investigated the interactions and combined effects of MSDP and genetic factors within a large prospective cohort, shedding light on the interplay between germline genetics and environmental factors in the progression of youth depression. However, our study faces several limitations. First, the GWAS findings have not yet been validated with external data, potentially affecting their generalizability. Additionally, as most UK Biobank participants are of White European ancestry, the generalizability of the genetic findings and MSDP effects to other populations may be limited. Second, both MSDP and youth depression were assessed retrospectively and may be subject to recall bias. Nevertheless, the UK Biobank MSDP measure has shown good concordance with biomarker-based indicators such as cotinine [72] and is widely used in large-scale epidemiologic studies [73]. Likewise, our youth depression definition incorporates multiple symptom-based items and age-of-onset reporting, and prior work indicates that retrospectively recalled depressive episodes reflect meaningful clinical severity [74]. Any remaining misclassification is expected to be nondifferential and would likely bias associations toward the null [75]. Third, the study did not distinguish between smoking exposures occurring at different stages of pregnancy or quantify exposure levels, nor did it assess the severity of youth depressive symptoms; therefore, a dose–response relationship could not be examined. In addition, although parental psychiatric histories were not directly obtained, we attempted to address genetic confounding using offspring genetic data. Importantly, while this study investigated the interaction between genetic susceptibility and MSDP in relation to youth depression, it did not extend this interaction analysis to adult physical health outcomes.

Utilizing a large prospective cohort study, we identified the *ABR* gene associated with youth depression. Moreover, we provided epidemiological evidence suggesting that MSDP and its interaction with genetic factors may increase the incidence of youth depression, particularly among females. This exposure could also lead to an increased prevalence of chronic physical comorbidities in young individuals with depression over the long term. The evidence robustly supports the implementation of targeted public health interventions for smoking cessation among expectant mothers. Preventing MSDP has the potential to significantly lower the incidence of mental and physical health challenges, markedly enhancing life quality and reducing long-term healthcare costs.

## Supplementary information

Supplementary Figure Legends; Supplementary Table 1-5.

Figure S1

Figure S2

## Data Availability

The genome-wide association study (GWAS) data for adult depression referenced in this study are available for download at the following link: https://figshare.com/articles/dataset/mdd2018/14672085?file=28169502.
