# Supplementary material for: Association of maternal smoking during pregnancy with youth depression and subsequent adult chronic diseases in offspring
Source: Transl Psychiatry. 2026 Mar 26;16:207. doi: 10.1038/s41398-026-03976-w (PMC13039871; doi:10.1038/s41398-026-03976-w)
Supplement: Supplementary file 1 — Supplementary Figure Legends; Supplementary Table 1-5. [file 41398_2026_3976_MOESM1_ESM.docx]

Supplementary Materials for:

**Association of Maternal Smoking During Pregnancy With Youth Depression and Subsequent Adult Chronic Diseases in Offspring**

**Supplementary Figure 1:** Schoenfeld Residuals for Covariates in Cox Proportional Hazards Model

**Supplementary Figure 2**: Gender-Specific GWAS Results for Youth Depression

**Supplementary Table 1**: Detailed ICD-10 Codes for Chronic Physical Diseases

**Supplementary Table 2:** Detailed Characteristics of Identified SNPs Associated with Youth Depression

**Supplementary Table 3:** Association of MSDP Exposure with Transitions from Youth Depression to Comorbid Chronic Conditions and Subsequent Mortality

**Supplementary Table** **4**: Association of MSDP Exposure with Transitions from Youth Depression to Comorbid Chronic Conditions and Subsequent Mortality in Female

**Supplementary Table** **5**: Association of MSDP Exposure with Transitions from Youth Depression to Comorbid Chronic Conditions and Subsequent Mortality in Male

**Supplementary Figure Legends**

**Figure S1.** **Schoenfeld Residuals for Covariates in Cox Proportional Hazards Model**

***Notes:*** Each panel displays scaled Schoenfeld residuals over time for one covariate: (A) MSPD, (B) Sex, (C) White Race, (D) Birthweight, (E) Body Size at Age 10, and (F) Breastfeeding. The solid red line represents the smoothed trend of the residuals, while the dashed line shows the 95% confidence interval. p-values are from the individual Schoenfeld tests for proportional hazards.

**Figure S2. Gender-Specific GWAS Results for Youth Depression**

***Notes:*** The top panel shows data for females and the bottom panel for males. Each dot represents a single nucleotide polymorphism (SNP), plotted with its negative logarithm of the p-value (-log10(p)) on the y-axis against its position across the chromosomes (Chr) on the x-axis. SNPs surpassing the genome-wide significance threshold (*P* < 5×10^-8^) are highlighted and labeled with their respective rs numbers.

**Table S1.** **Detailed ICD-10 Codes for Chronic Physical Diseases**

| **Condition** | **ICD-10 Codes** |
| --- | --- |
| Asthma | J45.x, J46.x |
| COPD | J44.x |
| Cardiac arrhythmia | I44.1-I44.3, I45.6, I45.9, I47.x-I49.x, R00.0, R00.1, R00.8, T82.1, Z45.0, Z95.0 |
| Congestive heart failure | I09.9, I11.0, I13.0, I13.2, I25.5, I42.0, I42.5-I42.9, I43.x, I50.x, P29.0 |
| Myocardial infarction | I21.x, I22.x, I25.2 |
| Cerebrovascular disease | G45.x, G46.x, H34.0, I60.x-I69.x |
| Neurological disorders | G10.x-G13.x, G20.x-G22.x, G25.4, G25.5, G31.2, G31.8, G31.9, G32.x, G35.x-G37.x, G40.x, G41.x, G93.1, G93.4, R47.0, R56.x |
| Diabetes (type 1 or 2, including with complications) | E10.0, E10.1, E10.9, E11.0, E11.1, E11.9, E12.0, E12.1, E12.9, E13.0, E13.1, E13.9, E14.0, E14.1, E14.9, E10.2-E10.8, E11.2-E11.8, E12.2-E12.8, E13.2-E13.8, E14.2-E14.8 |
| Hypothyroidism | E00.x-E03.x, E89.0 |
| Liver disease | B18.x, I85.x, I86.4, I98.2, K70.x, K71.1, K71.3-K71.5, K71.7, K72.x-K74.x, K76.0, K76.2-K76.9, Z94.4 |
| Renal disease | I12.0, I13.1, N03.2-N03.7, N05.2-N05.7, N18.x, N19.x, N25.0, Z49.0-Z49.2, Z94.0, Z99.2 |
| Peptic ulcers | K25.x-K28.x |
| Rheumatic and collagen disease | L94.0, L94.1, L94.3, M05.x, M06.x, M08.x, M12.0, M12.3, M30.x, M31.0-M31.3, M32.x-M35.x, M45.x, M46.1, M46.8, M46.9 |
| Paresis or paralysis | G04.1, G11.4, G80.1, G80.2, G81.x, G82.x, G83.0-G83.4, G83.9 |
| HIV/AIDS | B20.x-B22.x, B24.x |
| Hypertension | I10.x, I11.x-I13.x, I15.x |
| Peripheral vascular disease | I70.x, I71.x, I73.1, I73.8, I73.9, I77.1, I79.0, I79.2, K55.1, K55.8, K55.9, Z95.8, Z95.9 |
| Pulmonary circulation disorders | I26.x, I27.x, I28.0, I28.8, I28.9 |
| Valvular disease | A52.0, I05.x-I08.x, I09.1, I09.8, I34.x-I39.x, Q23.0-Q23.3, Z95.2-Z95.4 |
| Deficiency anaemia | D50.8, D50.9, D51.x-D53.x |
| Blood loss anaemia | D50.0 |
| Coagulopathy | D65-D68.x, D69.1, D69.3-D69.6 |
| Fluid or electrolyte disorders | E22.2, E86.x, E87.x |
| Cancer | C00.x-C26.x, C30.x-C34.x, C37.x-C41.x, C43.x, C45.x-C58.x, C60.x-C85.x, C88.x, C90.x-C97.x |

**Table S2. Detailed Characteristics of Identified SNPs Associated with Youth Depression**

| SNP | CHROM | POS | A1 | ALT | OR | SE | *P* | Nearest Gene |
| --- | --- | --- | --- | --- | --- | --- | --- | --- |
| rs112030396 | 17 | 1123047 | C | A | 1.31205 | 0.048895 | 2.78×10^-08^ | *ABR* |
| rs139138783 | 17 | 1123430 | C | A | 1.31297 | 0.048962 | 2.68×10^-08^ | *ABR* |
| rs144035576 | 17 | 1123441 | T | C | 1.31289 | 0.048962 | 2.70×10^-08^ | *ABR* |
| rs16943458 | 17 | 1121687 | T | G | 1.32022 | 0.048542 | 1.05×10^-08^ | *ABR* |
| rs74520265 | 17 | 1121522 | G | A | 1.32059 | 0.048463 | 9.58×10^-09^ | *ABR* |
| rs74878546 | 17 | 1121422 | T | C | 1.31375 | 0.048262 | 1.56×10^-08^ | *ABR* |
| rs75758739 | 17 | 1123697 | T | C | 1.31398 | 0.049016 | 2.54×10^-08^ | *ABR* |
| rs77028457 | 17 | 1122405 | T | C | 1.32466 | 0.048771 | 8.17×10^-09^ | *ABR* |
| rs78488970 | 17 | 1123069 | A | G | 1.31896 | 0.048826 | 1.43×10^-08^ | *ABR* |
| rs79326012 | 17 | 1122082 | G | A | 1.32421 | 0.048673 | 7.95×10^-09^ | *ABR* |

**Table S3. Association of MSDP Exposure with Transitions from Youth Depression to Comorbid Chronic Conditions and Subsequent Mortality**

| Comorbidity | MSDP → Youth depression | |  | MSDP → Death | |  | Youth depression → Comorbidity | |  | Youth depression → Death | |  | Comorbidity → Death | |
| --- | --- | --- | --- | --- | --- | --- | --- | --- | --- | --- | --- | --- | --- | --- |
|  | Case/Control | HR (95%CI) |  | Case/Control | HR (95%CI) |  | Case/Control | HR (95%CI) |  | Case/Control | HR (95%CI) |  | Case/Control | HR (95%CI) |
| Asthma | 3997/29721 | 1.26 (1.18-1.35) * |  | 325/33334 | 1.56 (1.23–1.97) * |  | 462/3525 | 1.37 (1.14–1.66) * |  | 27/3970 | 1.47 (0.66–3.26) |  | 8/406 | 0.32 (0.07–1.42) |
| Blood loss anaemia |  |  |  |  |  |  | 15/3972 | 1.84 (0.66–5.14) |  | 35/3962 | 1.38 (0.69–2.77) |  | 0/13 | - |
| COPD |  |  |  |  |  |  | 75/3912 | 3.53 (2.20–5.65) * |  | 34/3963 | 1.29 (0.63–2.63) |  | 1/53 | - |
| Cancer |  |  |  |  |  |  | 410/3577 | 0.88 (0.71–1.09) |  | 13/3984 | 1.37 (0.44–4.27) |  | 22/301 | 1.46 (0.58–3.65) |
| Cardiac arrhythmia |  |  |  |  |  |  | 296/3691 | 1.24 (0.97–1.57) |  | 26/3971 | 1.06 (0.46–2.48) |  | 9/216 | 2.13 (0.51–8.88) |
| Cerebrovascular disease |  |  |  |  |  |  | 108/3879 | 0.89 (0.58–1.35) |  | 31/3966 | 1.35 (0.64–2.86) |  | 4/62 | 0.07 (0.01–0.69) |
| Coagulopathy |  |  |  |  |  |  | 46/3941 | 1.02 (0.54–1.92) |  | 29/3968 | 1.57 (0.74–3.32) |  | 6/30 | - |
| Congestive heart failure |  |  |  |  |  |  | 65/3922 | 1.87 (1.14–3.06) |  | 31/3966 | 1.33 (0.63–2.82) |  | 4/42 | 0.17 (0.00–7.28) |
| Deficiency anaemia |  |  |  |  |  |  | 157/3830 | 1.11 (0.79–1.55) |  | 32/3965 | 1.40 (0.68–2.91) |  | 3/119 | - |
| Diabetes |  |  |  |  |  |  | 197/3790 | 1.44 (1.08–1.92) |  | 26/3971 | 1.74 (0.79–3.83) |  | 9/151 | 0.09 (0.01–0.95) |
| Fluid or electrolyte disorders |  |  |  |  |  |  | 140/3847 | 1.00 (0.70–1.43) |  | 26/3971 | 1.31 (0.57–2.99) |  | 9/71 | 1.59 (0.37–6.94) |
| HIV/AIDS |  |  |  |  |  |  | 5/3982 | 4.29 (0.71–26.03) |  | 34/3963 | 1.27 (0.62–2.60) |  | 1/4 | - |
| Hypertension |  |  |  |  |  |  | 794/3193 | 1.29 (1.12–1.50) * |  | 19/3978 | 1.34 (0.52–3.45) |  | 16/628 | 0.8 (0.28–2.27) |
| Hypothyroidism |  |  |  |  |  |  | 261/3726 | 1.10 (0.85–1.43) |  | 28/3969 | 1.62 (0.75–3.53) |  | 7/216 | 0.8 (0.15–4.29) |
| Liver disease |  |  |  |  |  |  | 119/3868 | 1.88 (1.31–2.71) * |  | 29/3968 | 1.51 (0.71–3.23) |  | 6/73 | 0.34 (0.05–2.21) |
| Myocardial infarction |  |  |  |  |  |  | 89/3898 | 1.24 (0.80–1.92) |  | 32/3965 | 1.61 (0.79–3.30) |  | 3/65 | - |
| Neurological disorders |  |  |  |  |  |  | 115/3872 | 0.98 (0.66–1.45) |  | 32/3965 | 1.26 (0.60–2.64) |  | 3/92 | 1.02 (0.09–11.64) |
| Paresis or paralysis |  |  |  |  |  |  | 23/3964 | 1.54 (0.67–3.54) |  | 35/3962 | 1.38 (0.69–2.77) |  | 0/17 | - |
| Peptic ulcers |  |  |  |  |  |  | 72/3915 | 1.18 (0.72–1.93) |  | 35/3962 | 1.40 (0.70–2.82) |  | 0/63 | - |
| Peripheral vascular disease |  |  |  |  |  |  | 74/3913 | 2.33 (1.47–3.71) * |  | 32/3965 | 1.33 (0.64–2.74) |  | 3/49 | - |
| Pulmonary circulation disorders |  |  |  |  |  |  | 55/3932 | 1.13 (0.64–2.00) |  | 31/3966 | 1.47 (0.70–3.05) |  | 4/39 | - |
| Renal disease |  |  |  |  |  |  | 106/3881 | 0.95 (0.63–1.44) |  | 30/3967 | 1.39 (0.65–2.96) |  | 5/65 | 1.11 (0.08–14.65) |
| Rheumatic and collagen disease |  |  |  |  |  |  | 143/3844 | 1.27 (0.90–1.79) |  | 34/3963 | 1.41 (0.70–2.84) |  | 1/121 | - |
| Valvular disease |  |  |  |  |  |  | 109/3878 | 0.84 (0.55–1.28) |  | 31/3966 | 1.52 (0.73–3.16) |  | 4/76 | 0.84 (0.07–9.88) |
| First disease | 2113/29721 | 1.28 (1.16-1.40) **^#^** |  | 325/31453 | 1.56 (1.23–1.97) **^#^** |  | 1580/526 | 1.25 (1.12–1.38) **^#^** |  | 0/2113 | - |  | 35/1545 | 1.08 (0.54–2.15) |
| Second disease | 3997/29721 | 1.26 (1.18-1.35) **^#^** |  | 325/33334 | 1.56 (1.23–1.97) **^#^** |  | 775/3212 | 1.20 (1.03–1.39) **^#^** |  | 5/3992 | 0.68 (0.07–6.14) |  | 30/745 | 1.14 (0.54–2.39) |
| Third disease | 3997/29721 | 1.26 (1.18-1.35) **^#^** |  | 325/33334 | 1.56 (1.23–1.97) **^#^** |  | 354/3633 | 1.38 (1.11–1.71) **^#^** |  | 15/3982 | 1.23 (0.42–3.67) |  | 20/334 | 1.02 (0.40–2.60) |
| Fourth disease | 3997/29721 | 1.26 (1.18-1.35) **^#^** |  | 325/33334 | 1.56 (1.23–1.97) **^#^** |  | 172/3815 | 1.45 (1.06–1.97) **^#^** |  | 22/3975 | 1.99 (0.85–4.67) |  | 13/159 | 0.25 (0.05–1.21) |
| Fifth disease | 3997/29721 | 1.26 (1.18-1.35) **^#^** |  | 325/33334 | 1.56 (1.23–1.97) **^#^** |  | 90/3897 | 1.51 (0.99–2.32) |  | 26/3971 | 1.51 (0.68–3.37) |  | 9/81 | 0.51 (0.08–3.36) |

***Notes***: The transitions “MSDP → Youth depression” and “MSDP → Death” do not vary by comorbidity. The full estimates are reported once in the table. COPD, chronic obstructive pulmonary disease. *, *P* < 0.0021. ^#^, *P* < 0.05.

**Table S4. Association of MSDP Exposure with Transitions from Youth Depression to Comorbid Chronic Conditions and Subsequent Mortality in Female**

| Comorbidity | MSDP →Youth depression | |  | MSDP → Death | |  | Youth depression → Comorbidity | |  | Youth depression → Death | |  | Comorbidity → Death | |
| --- | --- | --- | --- | --- | --- | --- | --- | --- | --- | --- | --- | --- | --- | --- |
|  | case/control | HR (95%CI) |  | case/control | HR (95%CI) |  | case/control | HR (95%CI) |  | case/control | HR (95%CI) |  | case/control | HR (95%CI) |
| Asthma | 2918 / 16009 | 1.34 (1.24-1.45) * |  | 137 / 18758 | 1.61 (1.12-2.32) |  | 359 / 2553 | 1.34 (1.08-1.66) |  | 17 / 2895 | 2.04 (0.74-5.63) |  | 7 / 314 | 0.31 (0.06-1.56) |
| Blood loss anaemia |  |  |  |  |  |  | 13 / 2899 | 2.35 (0.78-7.11) |  | 24 / 2888 | 1.79 (0.77-4.14) |  | 0 / 12 | - |
| COPD |  |  |  |  |  |  | 57 / 2855 | 3.16 (1.85-5.40) * |  | 23 / 2889 | 1.61 (0.68-3.82) |  | 1 / 38 | - |
| Cancer |  |  |  |  |  |  | 304 / 2608 | 0.85 (0.66-1.10) |  | 8 / 2904 | 2.40 (0.54-10.62) |  | 16 / 230 | 2.01 (0.68-5.94) |
| Cardiac arrhythmia |  |  |  |  |  |  | 207 / 2705 | 1.38 (1.04-1.83) |  | 16 / 2896 | 1.22 (0.41-3.63) |  | 8 / 145 | 2.37 (0.48-11.64) |
| Cerebrovascular disease |  |  |  |  |  |  | 69 / 2843 | 0.92 (0.55-1.54) |  | 20 / 2892 | 1.80 (0.71-4.61) |  | 4 / 34 | 0.07 (0.00-9.29) |
| Coagulopathy |  |  |  |  |  |  | 36 / 2876 | 1.32 (0.66-2.63) |  | 20 / 2892 | 1.88 (0.76-4.66) |  | - | - |
| Congestive heart failure |  |  |  |  |  |  | 39 / 2873 | 2.25 (1.18-4.27) |  | 21 / 2891 | 1.60 (0.64-4.00) |  | 3/24 | - |
| Deficiency anaemia |  |  |  |  |  |  | 130 / 2782 | 1.19 (0.83-1.70) |  | 21 / 2891 | 1.95 (0.79-4.80) |  | 3 / 101 | - |
| Diabetes |  |  |  |  |  |  | 119 / 2793 | 1.37 (0.94-1.98) |  | 17 / 2895 | 2.26 (0.83-6.15) |  | 7 / 87 | 0.04 (0.00-1.77) |
| Fluid or electrolyte disorders |  |  |  |  |  |  | 101 / 2811 | 0.97 (0.64-1.49) |  | 17 / 2895 | 2.01 (0.72-5.63) |  | 7 / 46 | 0.89 (0.13-6.01) |
| HIV_AIDS |  |  |  |  |  |  | - | - |  | 24 / 2888 | 1.79 (0.77-4.13) |  | - | - |
| Hypertension |  |  |  |  |  |  | 534 / 2378 | 1.32 (1.11-1.58) * |  | 11 / 2901 | 1.43 (0.40-5.09) |  | 13 / 412 | 1.18 (0.38-3.71) |
| Hypothyroidism |  |  |  |  |  |  | 235 / 2677 | 1.12 (0.85-1.47) |  | 18 / 2894 | 2.43 (0.90-6.56) |  | 6 / 196 | 1.07 (0.19-5.97) |
| Liver disease |  |  |  |  |  |  | 85 / 2827 | 1.79 (1.16-2.76) |  | 19 / 2893 | 2.51 (0.99-6.39) |  | 5 / 48 | 0.15 (0.01-1.62) |
| Myocardial infarction |  |  |  |  |  |  | 42 / 2870 | 0.96 (0.50-1.87) |  | 22 / 2890 | 2.11 (0.89-5.02) |  | 2/28 | - |
| Neurological disorders |  |  |  |  |  |  | 83 / 2829 | 0.92 (0.57-1.46) |  | 22 / 2890 | 1.47 (0.60-3.62) |  | 2 / 64 | - |
| Paresis or paralysis |  |  |  |  |  |  | 14 / 2898 | 1.92 (0.67-5.52) |  | 24 / 2888 | 1.79 (0.77-4.13) |  | 0 / 8 | - |
| Peptic ulcers |  |  |  |  |  |  | 43 / 2869 | 0.89 (0.45-1.75) |  | 24 / 2888 | 1.79 (0.78-4.15) |  | 0 / 39 | - |
| Peripheral vascular disease |  |  |  |  |  |  | 42 / 2870 | 2.63 (1.41-4.90) |  | 22 / 2890 | 1.92 (0.81-4.53) |  | 2/28 | - |
| Pulmonary circulation disorders |  |  |  |  |  |  | 41 / 2871 | 1.34 (0.70-2.55) |  | 20 / 2892 | 2.09 (0.84-5.22) |  | 4/26 | - |
| Renal disease |  |  |  |  |  |  | 78 / 2834 | 0.86 (0.52-1.40) |  | 21 / 2891 | 1.99 (0.81-4.93) |  | 3 / 47 | - |
| Rheumatic and collagen disease |  |  |  |  |  |  | 116 / 2796 | 1.43 (0.98-2.09) |  | 23 / 2889 | 1.83 (0.78-4.28) |  | 1 / 97 | - |
| Valvular disease |  |  |  |  |  |  | 71 / 2841 | 0.73 (0.43-1.25) |  | 21 / 2891 | 1.96 (0.80-4.79) |  | 3 / 49 | 2.19 (0.17-28.85) |
| First disease | 1512 / 16009 | 1.33 (1.19-1.49) ^#^ |  | 137 / 17354 | 1.61 (1.12-2.32) ^#^ |  | 1149 / 359 | 1.24 (1.09-1.40) ^#^ |  | 0 / 1508 | - |  | 24 / 1125 | 1.45 (0.63-3.31) |
| Second disease | 2918 / 16009 | 1.34 (1.24-1.45) ^#^ |  | 137 / 18758 | 1.61 (1.12-2.32) ^#^ |  | 541 / 2371 | 1.21 (1.02-1.45) ^#^ |  | 2 / 2910 | 5.41 (0.34-86.54) |  | 22 / 519 | 1.13 (0.47-2.73) |
| Third disease | 2918 / 16009 | 1.34 (1.24-1.45) ^#^ |  | 137 / 18758 | 1.61 (1.12-2.32) ^#^ |  | 246 / 2666 | 1.34 (1.03-1.74) |  | 9 / 2903 | 1.30 (0.31-5.41) |  | 15 / 231 | 1.35 (0.46-3.97) |
| Fourth disease | 2918 / 16009 | 1.34 (1.24-1.45) ^#^ |  | 137 / 18758 | 1.61 (1.12-2.32) ^#^ |  | 112 / 2800 | 1.51 (1.03-2.21) |  | 13 / 2899 | 3.04 (0.96-9.61) |  | 11 / 101 | 0.32 (0.06-1.62) |
| Fifth disease | 2918 / 16009 | 1.34 (1.24-1.45) ^#^ |  | 137 / 18758 | 1.61 (1.12-2.32) ^#^ |  | 56 / 2856 | 2.01 (1.18-3.43) |  | 16 / 2896 | 2.08 (0.73-5.91) |  | 8 / 48 | 0.54 (0.08-3.81) |

***Notes***: The transitions “MSDP → Youth depression” and “MSDP → Death” do not vary by comorbidity. The full estimates are reported once in the table. COPD, chronic obstructive pulmonary disease. *, *P* < 0.0021. ^#^, *P* < 0.05.

**Table S5. Association of MSDP Exposure with Transitions from Youth Depression to Comorbid Chronic Conditions and Subsequent Mortality in Male**

| Comorbidity | MSDP →Youth depression | |  | MSDP → Death | |  | Youth depression → Comorbidity | |  | Youth depression → Death | |  | Comorbidity → Death | |
| --- | --- | --- | --- | --- | --- | --- | --- | --- | --- | --- | --- | --- | --- | --- |
|  | case/control | HR (95%CI) |  | case/control | HR (95%CI) |  | case/control | HR (95%CI) |  | case/control | HR (95%CI) |  | case/control | HR (95%CI) |
| Asthma | 1079/13712 | 1.06 (0.93-1.22) |  | 188/14576 | 1.52 (1.12-2.07) |  | 103/972 | 1.47 (0.98-2.20) |  | 10/1065 | 1.11 (0.28-4.43) |  | - | - |
| Blood loss anaemia |  |  |  |  |  |  | 2/1073 | - |  | 11/1064 | 0.92 (0.24-3.52) |  | - | - |
| COPD |  |  |  |  |  |  | 18/1057 | 5.10 (1.88-13.84) * |  | 11/1064 | 0.90 (0.23-3.49) |  | - | - |
| Cancer |  |  |  |  |  |  | 106/969 | 0.92 (0.60-1.41) |  | 5/1070 | 0.53 (0.06-4.91) |  | 6/71 | 0.26 (0.03-2.32) |
| Cardiac arrhythmia |  |  |  |  |  |  | 89/986 | 0.95 (0.60-1.51) |  | 10/1065 | 1.00 (0.25-3.96) |  | - | - |
| Cerebrovascular disease |  |  |  |  |  |  | 39/1036 | 0.81 (0.39-1.67) |  | 11/1064 | 0.99 (0.26-3.78) |  | - | - |
| Coagulopathy |  |  |  |  |  |  | 10/1065 | 0.26 (0.03-2.03) |  | 9/1066 | 1.26 (0.31-5.12) |  | 2/7 | - |
| Congestive heart failure |  |  |  |  |  |  | 26/1049 | 1.43 (0.65-3.17) |  | 10/1065 | 1.11 (0.28-4.33) |  | - | - |
| Deficiency anaemia |  |  |  |  |  |  | 27/1048 | 0.69 (0.28-1.73) |  | 11/1064 | 0.90 (0.24-3.48) |  | - | - |
| Diabetes |  |  |  |  |  |  | 78/997 | 1.51 (0.95-2.40) |  | 9/1066 | 1.38 (0.34-5.58) |  | 2/64 | - |
| Fluid or electrolyte disorders |  |  |  |  |  |  | 39/1036 | 1.08 (0.54-2.14) |  | 9/1066 | 0.68 (0.14-3.41) |  | 2/25 | - |
| HIV_AIDS |  |  |  |  |  |  | 5/1070 | 4.29 (0.71-26.03) |  | 10/1065 | 0.63 (0.13-3.06) |  | - | - |
| Hypertension |  |  |  |  |  |  | 260/815 | 1.25 (0.96-1.62) |  | 8/1067 | 1.42 (0.33-6.06) |  | 3/216 | - |
| Hypothyroidism |  |  |  |  |  |  | 26/1049 | 0.92 (0.39-2.19) |  | 10/1065 | 1.00 (0.25-4.01) |  | - | - |
| Liver disease |  |  |  |  |  |  | 34/1041 | 2.27 (1.15-4.47) |  | 10/1065 | 0.65 (0.13-3.13) |  | - | - |
| Myocardial infarction |  |  |  |  |  |  | 47/1028 | 1.55 (0.86-2.79) |  | 10/1065 | 1.13 (0.29-4.40) |  | - | - |
| Neurological disorders |  |  |  |  |  |  | 32/1043 | 1.26 (0.61-2.64) |  | 10/1065 | 1.12 (0.29-4.40) |  | - | - |
| Paresis or paralysis |  |  |  |  |  |  | 9/1066 | 1.06 (0.26-4.35) |  | 11/1064 | 0.92 (0.24-3.53) |  | - | - |
| Peptic ulcers |  |  |  |  |  |  | 29/1046 | 1.77 (0.83-3.76) |  | 11/1064 | 0.96 (0.25-3.71) |  | - | - |
| Peripheral vascular disease |  |  |  |  |  |  | 32/1043 | 1.93 (0.95-3.92) |  | 10/1065 | 0.56 (0.11-2.77) |  | - | - |
| Pulmonary circulation disorders |  |  |  |  |  |  | 14/1061 | 0.68 (0.19-2.46) |  | 11/1064 | 0.91 (0.24-3.52) |  | - | - |
| Renal disease |  |  |  |  |  |  | 28/1047 | 1.15 (0.51-2.59) |  | 9/1066 | 0.72 (0.15-3.52) |  | 2/18 | - |
| Rheumatic and collagen disease |  |  |  |  |  |  | 27/1048 | 0.68 (0.27-1.70) |  | 11/1064 | 0.92 (0.24-3.53) |  | - | - |
| Valvular disease |  |  |  |  |  |  | 38/1037 | 1.06 (0.53-2.13) |  | 10/1065 | 1.13 (0.29-4.43) |  | - | - |
| First disease | 601/13712 | 1.15 (0.96-1.37) |  | 188/14099 | 1.52 (1.12-2.07) ^#^ |  | 431/167 | 1.22 (0.99-1.50) |  | - | - |  | 11/420 | 0.70 (0.18-2.68) |
| Second disease | 1079/13712 | 1.06 (0.93-1.22) |  | 188/14576 | 1.52 (1.12-2.07) ^#^ |  | 234/841 | 1.17 (0.88-1.54) |  | 3/1072 | - |  | 8/226 | 1.49 (0.34-6.57) |
| Third disease | 1079/13712 | 1.06 (0.93-1.22) |  | 188/14576 | 1.52 (1.12-2.07) ^#^ |  | 108/967 | 1.50 (1.01-2.22) ^#^ |  | 6/1069 | 1.42 (0.25-7.97) |  | 5/103 | 0.50 (0.05-4.74) |
| Fourth disease | 1079/13712 | 1.06 (0.93-1.22) |  | 188/14576 | 1.52 (1.12-2.07) ^#^ |  | 60/1015 | 1.37 (0.80-2.33) |  | 9/1066 | 1.39 (0.34-5.64) |  | 2/58 | - |
| Fifth disease | 1079/13712 | 1.06 (0.93-1.22) |  | 188/14576 | 1.52 (1.12-2.07) ^#^ |  | 34/1041 | 0.89 (0.42-1.92) |  | 10/1065 | 1.14 (0.29-4.48) |  | - | - |

***Notes***: The transitions “MSDP → Youth depression” and “MSDP → Death” do not vary by comorbidity. The full estimates are reported once in the table. COPD, chronic obstructive pulmonary disease. *, *P* < 0.0021. ^#^, *P* < 0.05
