# Supplementary figures and images for: Association of maternal smoking during pregnancy with youth depression and subsequent adult chronic diseases in offspring
Source: Transl Psychiatry. 2026 Mar 26;16:207. doi: 10.1038/s41398-026-03976-w (PMC13039871; doi:10.1038/s41398-026-03976-w)

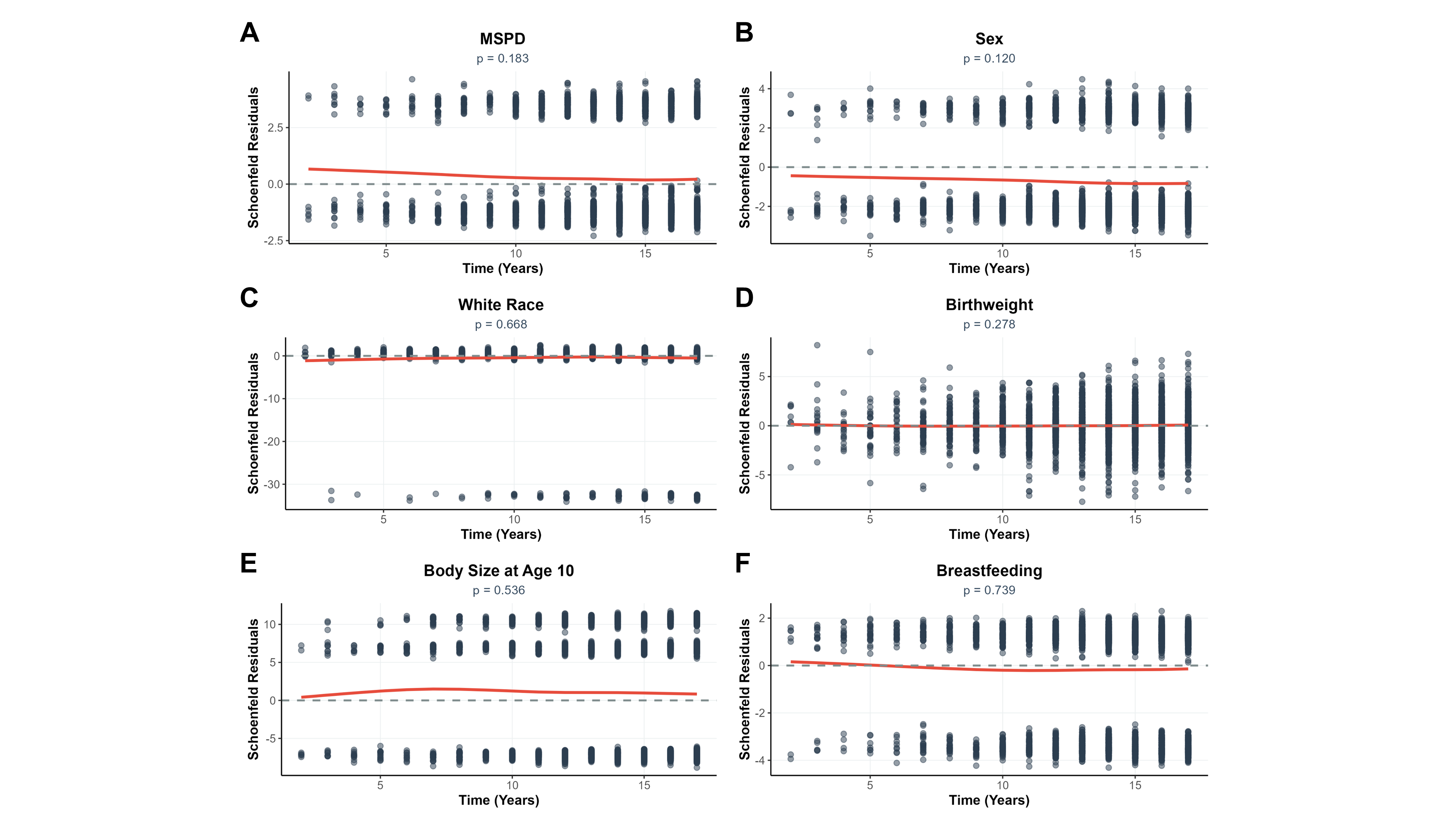

Supplement: Supplementary file 2 — Figure S1 [file 41398_2026_3976_MOESM2_ESM.tif]

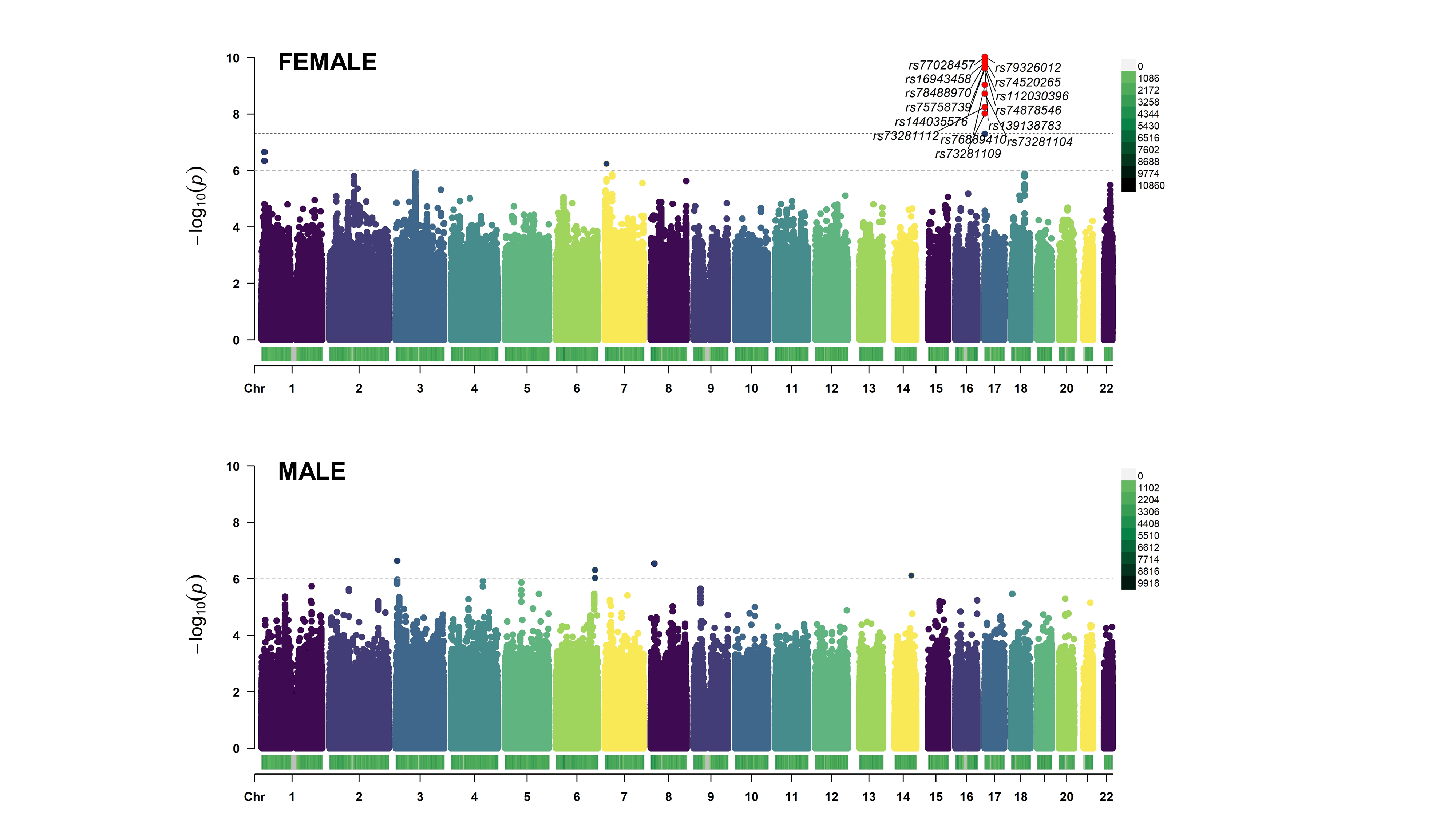

Supplement: Supplementary file 3 — Figure S2 [file 41398_2026_3976_MOESM3_ESM.tif]
